# Wavelet Cross-Correlation to Investigate Regional Variations in Cerebral Oxygenation in Infants Supported on Extracorporeal Membrane Oxygenation

**DOI:** 10.1007/978-1-4614-4989-8_28

**Published:** 2012-07-21

**Authors:** Maria Papademetriou, Ilias Tachtsidis, Martin J. Elliott, Aparna Hoskote, Clare E. Elwell

**Affiliations:** 1 Medical Physics and Bioengineering Department, University College London, Malet Place Engineering Building, Gower Street, London, WC1E 6BT UK; 2 Cardiothoracic Unit, Great Ormond Street Hospital for Children, London, UK

**Keywords:** Cerebral oxygenation, Infants

## Abstract

Extracorporeal membrane oxygenation can potentially affect cerebral blood flow dynamics and consequently influence cerebral autoregulation. We applied wavelet cross-correlation (WCC) between multichannel cerebral oxyhemoglobin concentration (HbO_2_) and mean arterial pressure (MAP), to assess regional variations in cerebral autoregulation. Six infants on veno-arterial (VA) ECMO were studied during sequential changes in the ECMO flows. WCC between MAP and HbO_2_ for each flow period and each channel was calculated within three different frequency (wavelet scale) bands centered around 0.1, 0.16, and 0.3 Hz chosen to represent low frequency oscillations, ventilation, and respiration rates, respectively. The group data showed a relationship between maximum WCC and ECMO flow. During changes in ECMO flow, statistically significant differences in maximum WCC were found between right and left hemispheres. WCC between HbO_2_ and MAP provides a useful method to investigate the dynamics of cerebral autoregulation during ECMO. Manipulations of ECMO flows are associated with regional changes in cerebral autoregulation which may potentially have an important bearing on clinical outcome.

## Introduction

Extracorporeal membrane oxygenation (ECMO) is a life support system for infants with cardiorespiratory failure. Neurological complications are the largest cause of morbidity and mortality in these patients, with the reported frequency of abnormal neuroimaging ranging from 28 to 52 % [1]. Initiation of ECMO involves cannulation of the major great vessels—right common carotid artery and internal jugular vein—which may cause lateralizing cerebrovascular injury. ECMO infants suffer from hypoxia, asphyxia, and hypercarbia which can disrupt cerebral autoregulation, leaving the cerebral microcirculation vulnerable to alterations in blood pressure [2].

Methods to assess the status of autoregulation by considering the relationship between spontaneous fluctuations in MAP and cerebral blood flow (CBF) surrogates, such as (HbO_2_) measured by NIRS, in either the time or frequency domain using Fourier transforms were reported extensively in the literature [3]. These, conventional methods suffer from the big drawback of averaging out all the potential useful time information, hence treating cerebral autoregulation as a stationary, linear process.

Recent studies have emphasized that cerebral autoregulation is a dynamic process [4]. The continuous wavelet transform (CWT) possesses the ability to construct a time–frequency representation of a signal that offers time and frequency localization. Latka et al. used CWT to compute a synchronization index between CBF and ABP signals [5]. Wavelet cross-correlation (WCC) was introduced by Rowley et al. as the cross-correlation between CWT coefficients of two time series [6]. Spectral analysis using wavelets provides a framework for analysis of nonstationary effects in cerebral hemodynamics, thus overcoming the restrictions intrinsic to earlier methods.

Previously we used a dual-channel NIRS system and showed the presence of oscillations related to vasomotion, respiration, and heart rates [7]. Preliminary results using multichannel NIRS indicate regional variation in cerebral oxygenation [8]. Here, we investigate the use of WCC as a method to study the concordance between multisite cerebral HbO_2_ and mean arterial pressure in order to assess regional variations in cerebral oxygenation in neonates supported on ECMO.

## Methods

### Subjects and Instrumentation

A total of six veno-arterial (VA) ECMO patients, age range 1–16 days, were monitored during sequential changes in the ECMO flows. Alterations in the ECMO flows refer to successive decrease in the ECMO flow by 10 % from the initial flow, approximately every 10 min, down to 70 % of the initial flow followed by successive increase back to baseline (Fig. 28.2b).

A multichannel NIRS system (ETG-100, Hitachi Medical Ltd., Japan) was used to measure changes in oxy-(HbO_2_), deoxy-(HHb), and total hemoglobin (HbT) concentrations at 5 Hz. A novel cap was constructed to accommodate the optical sources and detectors (interoptode distance  =  3 cm), allowing data to be collected from 12 channels. Multimodal data were collected synchronously including systemic parameters (arterial blood pressure [ABP], heart rate [HR], and arterial oxygen saturation [SpO_2_]) and ECMO circuit parameters (venous oxygen saturation [SvO_2_], arterial saturation at the cannula [SaO_2_]).

### Data Analysis

Mean arterial pressure (MAP) was obtained by trapezoid integration of ABP every 0.2 s, equivalent to sampling frequency of 5 Hz. The time series of MAP and HbO_2_ were divided into sections representing each ECMO flow period (Fig 28.3c). Each section of data was then high and low pass filtered at 0.008 and 1 Hz.

An approximate relationship between the scale *α* in the wavelet domain and frequency in the Fourier transform, *f*_*a*_, can be computed as [5]:

28.1 $$ {f}_{\alpha }=\frac{{f}_{\text{c}}}{\alpha \cdot \delta t},$$

where *f*_c_ is the center frequency and δ*t* is the sampling period.

Wavelet analysis was performed on HbO_2_ data. WCC and synchronization index, *γ*, were used as methods to investigate the relation between MAP and HbO_2_. The complex Morlet wavelet was used to calculate the CWT coefficients for MAP and HbO_2_ using the MatLab wavelet toolbox function *cwt*. The central frequency (*f*_c_) and bandwidth (*f*_b_) of the complex Morlet wavelet were both chosen as 1 in order to be in agreement with previous methods [5, 6]. A scale range with unit spacing from 5 to 100, representing frequencies 0.008–1 Hz was used to obtain two complex time series, ${W}_{\text{MAP}}(a,t)$ and ${W}_{{\text{HbO}}_{2}}(a,t)$ for each flow period A–G and across each of the 12 channels.

The WCC between MAP and HbO_2_ in each channel and for each flow was obtained using the equation below [6]:

28.2 $$ \overline{\text{WCC}}=\frac{\left|{R}_{X,Y}({W}_{\text{MAP}},{W}_{{\text{HbO}}_{2}}\alpha,\tau )\right|}{\sqrt{\left|{R}_{X,X}({W}_{\text{MAP}},\alpha,0)\cdot {R}_{X,X}({W}_{{\text{HbO}}_{2}}\alpha,0)\right|}},$$

in which *R*_*X*,*Y*_(*s*1, *s*2, *a*, *τ*) denotes the cross-correlation of the wavelet coefficients of the series *s*1 and *s*2 at a scale *α* and for a relative time shift *τ* and *R*_*X*,*X*_(*s*1, *a*, 0) denotes the autocorrelation of the time series *s*1 for zero time shift. WCC(*a*, *τ*) represents the cross-spectral power in the two time series (shifted relative to each other by *τ*) as a fraction of the total power in the two time series. WCC ranges from 0 to 1. At a given wavelet scale, WCC  =  1 would indicate that the coefficients of the two wavelet transforms are related to each other by a simple scaling factor, suggesting strong synchronization at this frequency [6].

The phase difference between the two time series, MAP and HbO_2_ was also calculated using the circular mean, $\overline{\Delta \Phi (\alpha )}$, of the instantaneous phase difference between the two signals *ΔΦ*(*α*, *τ*) over the duration of a test segment [5]

28.3 $$ \overline{\Delta \Phi (\alpha )}={\mathrm{tan}}^{-1}\left(\frac{{\sum }_{t}\mathrm{sin}(\Delta \varphi (\alpha,t))}{{\sum }_{t}\mathrm{cos}(\Delta \varphi (\alpha,t))}\right).$$

For each time series pair at each flow period and for each channel, the maximum value of WCC(*a*, *τ*) was found within three scale bands: *a*_*i*_  =  5  *<  a  <*  20 (*f*_*ai*_  *=*  0.25 Hz  *<  f*_*a*_  *<  1*  Hz), *a*_*ii*_  =  20  *<  a  <*  40 (*f*_*aii*_  *=*  0.13 Hz  *<  f*_*a*_  *<*  0.25 Hz), *a*_*iii*_  =  40  *<  a  <*  80 (*f*_*aiii*_  *=*  0.06 Hz  *<  f*_*a*_  *<*  0.13 Hz). These bands were chosen to overlap with respiration rate (RR), ventilation rate (VR), and slow M-waves, respectively. The maximum circular mean, *ΔΦ*_max_, were also calculated within each scale band, for each flow period and each channel. Student’s  *t*-test was then used to analyze the statistical significance of the differences in the group mean of each of these variables between channels.

## Results

**Fig. 28.1:**
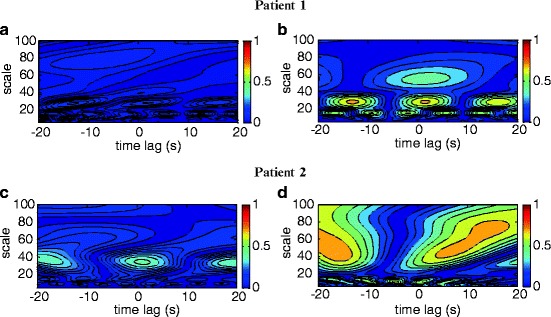
Wavelet cross-correlation (WCC) between MAP and HbO_2_ for two ECMO patients. Low correlation is shown at baseline ECMO flows (**a** and **c**) and high correlation around scales 16 and 30 for patient 1 at minimum flow (**b**) and around scales 16 and 40–80 for patient 2 (**d**) at minimum flow

Figure 28.1 shows a set of typical WCC contours obtained from two patients at baseline flow and minimum flow. For patient 1 WCC shows no distinct peaks at baseline flows indicating no correlation between MAP and HbO_2_. At minimum flow, peaks in the WCC contours are shown at scales 15 (*f*_*a*_  =  0.33 Hz), 29 (*f*_*a*_  =  0.17 Hz) and a relatively weaker peak at scale 55 (*f*_*a*_  =  0.09 Hz). WCC for patient 2 at baseline flow shows a relatively weak peak at a scale 34 (*f*_*a*_  =  0.15 Hz). As with patient 1 correlation between MAP and HbO_2_ becomes stronger at minimum flow with the peak at scale 34 spreading to higher Mayer-waves related scales and another peak occurring at scale 10 (*f*_*a*_  =  0.5 Hz). These peaks appear shifted from zero time lag in agreement with Rowley et al. [6].

In general, WCC between MAP and HbO_2_ revealed three distinct peaks within three scale regions. The first peak typically occurs at a scale of around 14 (0.36 Hz), the second at a scale around 30 (0.16 Hz) and the third at a scale around 50 (0.1 Hz). These peaks could correspond to the RR, VR, and Mayer-waves, respectively.

**Fig. 28.2:**
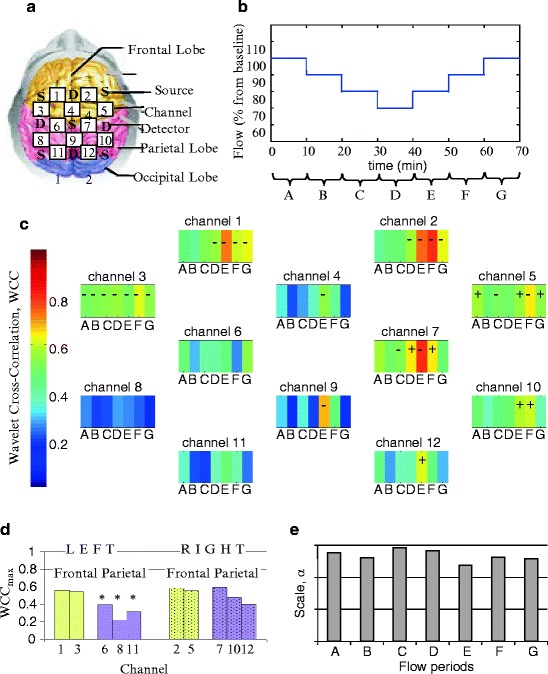
Group WCC on between MAP and HbO_2_ within scale band *a*_*i*_  =  5  <  *a*  <  20 ($$ {f}_{{a}_{i}}$$  =  0.25 Hz  <  *f*_*a*_  <  1 Hz): (**a**) channel arrangement; (**b**) sequence of flow changes (*A*  =  100 %, *B*  =  90 %, *C*  =  80 %, *D*  =  70 %, *E*  =  80 %, *F*  =  90 %, *G*  =  100 %); (**c**) $$ {\text{WCC}}_{\mathrm{max}}^{i}$$ at all flow periods across all channels; (**d**) mean $$ {\text{WCC}}_{\mathrm{max}}^{i}$$ across all flow periods of channels on the right side and symmetrical channels on the left side; (**e**) mean of scale at $$ {\text{WCC}}_{\mathrm{max}}^{i}$$, $$ {a}_{\mathrm{max}}^{i}$$, for each flow period across all channels. +/- denotes HbO_2_ lagging/leading MAP for $$ {\text{WCC}}_{\mathrm{max}}^{i}<0.5$$. (*asterisk*) Statistical significant difference between symmetrical channels on right and left hemispheres (*p*  <  0.05)

Figure 28.2 shows the group data for the mean of the maximum WCC, $$ {\text{WCC}}_{\mathrm{max}}^{i}$$, within scale band *a*_*i*_  =  *5  <  a  <*  20 ($$ {f}_{a}^{i}$$  *=*  0.25 Hz  *<  f*_*a*_  *<*  1 Hz) at each flow period and across the 12 channels. By convention a value of WCC below 0.5 indicates no correlation between MAP and HbO_2_ [5]. A + sign indicates that HbO_2_ lags MAP, i.e. Δ*Φ*  >  0, only for WCC  >  0.5. A – sign is used to indicate that HbO_2_ is leading MAP, i.e. Δ*Φ*  <  0, where WCC  >  0.5. There are statistically significant differences (*p*  <  0.05) in mean $$ {\text{WCC}}_{\mathrm{max}}^{i}$$ across all flows between symmetrical channels most likely positioned on the right and left parietal lobes (Fig. 28.3d). $$ {\text{WCC}}_{\mathrm{max}}^{i}$$ for all flows in the three channels positioned on the left parietal lobe is below 0.5 suggesting no correlation between MAP and HbO_2_ in these channels. A general increase in $$ {\text{WCC}}_{\mathrm{max}}^{i}$$was observed with decrease in flow across all channels. $$ {\text{WCC}}_{\mathrm{max}}^{i}$$ is highest either at flow period E or F. $$ {a}_{\mathrm{max}}^{i}$$ across flow changes for all channels ranges from 9 to 17 (0.29–0.56 Hz) (Fig. 28.3e). Most of the channels show a shift in $$ {a}_{\mathrm{max}}^{i}$$ to a lower scale when the highest $$ {\text{WCC}}_{\mathrm{max}}^{i}$$ is reached (flow period E).

## Discussion and Conclusions

WCC between HbO_2_ and MAP provides a useful method to investigate the dynamics of cerebral autoregulation. Cerebral autoregulation on ECMO is poorly studied, since there have been no easy noninvasive methods to study and interpret complex cerebral physiological process. Our results showed a relationship between WCC and ECMO flow in the grouped data of six patients. These differences were statistically significant between right and left hemispheres, especially when the flows were weaned sequentially. Modest manipulations of ECMO flows are associated with regional changes in cerebral autoregulation which may potentially have an important bearing on clinical outcome.
